# Molecular characterisation of triple negative essential thrombocythaemia patients by platelet analysis and targeted sequencing

**DOI:** 10.1038/bcj.2016.75

**Published:** 2016-08-26

**Authors:** A Angona, C Fernández-Rodríguez, A Alvarez-Larrán, L Camacho, R Longarón, E Torres, S Pairet, C Besses, B Bellosillo

**Affiliations:** 1 Department of Haematology, Hospital del Mar, Barcelona, Spain; 2 Universitat Autònoma de Barcelona, Barcelona, Spain; 3 Grup de Recerca Clínica Aplicada en Neoplàsies Hematològiques-IMIM (Hospital del Mar Medical Research Institute), Barcelona, Spain; 4 Department of Pathology, Hospital del Mar, Barcelona, Spain; 5 Universitat Pompeu Fabra, Barcelona, Spain

Essential thrombocythaemia (ET) is a myeloproliferative neoplasm (MPN) characterised by megakaryocyte hyperplasia and thrombocytosis. From the genetic perspective, ET patients harbour mutations in *JAK2* (50–60%), *CALR* (15–30%) and *MPL* (1–5%) genes.^1^ These frequencies have been determined in most studies using DNA obtained from isolated granulocytes or from whole blood, but, there is little information regarding the prevalence of these mutations when platelets are analysed. Some previous studies have reported that in ~10% of ET patients the *JAK2*V617F mutation was only detectable when platelets are analysed, in agreement with the predominant involvement of the megakaryocytic-platelet lineage in this disease.^2, 3^ In contrast, some studies suggested no differences in the frequency of *JAK2*V617F mutation between platelets and granulocytes when allele specific RT-PCR and RFLP analysis of platelet RNA was performed.^4, 5^ In this sense, our group has observed a higher *JAK2*V617F allele burden in platelets when compared with granulocytes in ET patients, although no differences were found in the number of mutated cases.^6^ Concerning mutations in *MPL* gene, the analysis of 82 ET patients showed in two cases the presence of *MPL*W515L in platelets but not in granulocytes, T-cells or erythroid colonies.^3^ Finally, there is scarce information regarding the presence of *CALR* mutations in platelets.

Overall, there is general agreement that 10–30% of all ET patients are wild type for *JAK2*, *CALR* and *MPL* mutations when the molecular analyses are performed with DNA from isolated granulocytes or peripheral blood.^1^ This subgroup of patients, called ‘triple-negative' (TN), has not been extensively studied with regard to the presence of *JAK2*, *CALR* and *MPL* mutations in platelets and RNA from granulocytes.

Recently, atypical mutations of *MPL* and *JAK2* were identified by whole-exome sequencing in a proportion of TN ET patients (10–20%), suggesting that this group of patients represent a heterogeneous disease category.^7, 8^

We have characterised the molecular profile of a group of TN ET patients by determining *JAK2*, *CALR* and *MPL* mutational profile using RNA from platelets. Furthermore, we have assessed the presence of additional mutations in *JAK2* and *MPL* and other non-driver genes by targeted next-generation sequencing (NGS) in granulocytes.

A total of 35 triple negative ET patients (14.8% from the whole cohort of 236 ET) diagnosed at the Haematology Department from the Hospital del Mar were included in the study. The diagnosis of ET was established according to WHO criteria.^9^ At the time when platelet analysis was performed patients were not receiving citoreductive therapy. The study was approved by the local Ethics Committee and informed consent was provided according to the Declaration of Helsinki.

All patients had been routinely assessed for mutations in DNA from purified granulocytes. *JAK2*V617F was determined by quantitative allele-specific PCR, *MPL* exon 10 mutations (W515, S505) were analysed by Sanger sequencing and *CALR* exon 9 mutations were analysed by PCR followed by fragment analysis as previously described.^10, 11, 12^

RNA was extracted from platelets or granulocytes with Trizol (Life Technologies, Carlsbad, CA) and 1 ug total RNA was reverse transcribed. The mutational analysis of *JAK2*V617F was performed by quantitative allele-specific real time PCR with probes specific for the mutated and the wild-type form as described previously.^10^ Analysis of exon 10 of the *MPL* gene (S505, W515) was performed by NGS (454 GS Junior, Roche Applied Science, Mannheim, Germany), with a median coverage of 1335x (range 497–4863). Mutations were confirmed by competitive allele specific TaqMan (CAST)-PCR assays (Life Technologies). The mutational analysis of exon 9 of the *CALR* gene was performed by PCR, using a 6-carboxyfluorescein labelled reverse primer, followed by fragment analysis in a Genetic Analyser 3500DX (Applied Biosystems, Foster, CA, USA) or by NGS deep sequencing (454 GS Junior, Roche Applied Science) with a median coverage of 1326.5x (range 607–1686). In those patients in whom a mutation was observed in platelets, we extracted RNA from granulocytes to assess the presence of the mutation in these cells.

Screening for additional somatic mutations was performed by targeted NGS in DNA extracted from purified granulocytes. All mutations detected were confirmed by Sanger sequencing. Clonality based on X chromosome inactivation pattern by *HUMARA* was also analysed.

The main clinical and biological characteristics of the patients are shown in Table 1. As can been seen, with a median follow-up of 7 years (range: 0–27), 4 (11.4%) patients presented thrombotic events and 4 (11.4%) haemorrhagic events during the evolution. No cases of transformation to acute leukaemia or secondary myelofibrosis occurred.

Platelet analysis of *JAK2, MPL and CALR* showed the presence of *JAK2V617F* in 2 out of 35 (5.7%) patients analysed, with allele burdens of 16 and 20%. Regarding mutations in *MPL* gene, analysis of exon 10 by NGS showed *MPL*W515L in three cases with an allele burden of 2.16, 12 and 26% in platelets (Table 2). These results were confirmed by independent PCR amplifications and NGS sequencing runs. Interestingly, we detected the coexistence of *JAK2*V617F and *MPL*W515L in two cases. No mutations in *CALR* were detected in the analysis of platelet RNA by fragment analysis or NGS.

Next, we also assessed whether these mutations could be detected in granulocytic RNA. We demonstrated the presence of three of these five mutations also in granulocytes when RNA was analysed (Table 2). These results suggest that RNA expression of mutant cells is higher than that from wild type cells, thus improving the detection rate, when compared with analysis of DNA. Furthermore, analysis of granulocyte DNA using commercial CAST-PCR assays that increase mutation detection by blocking amplification of the wild type allele, confirmed the presence of the *MPL* mutation in granulocytes in the two patients with higher allele burden. In the same way, applying Larsen assay in granulocytic DNA,^13^ we confirmed *JAK2*V617F in the two patients with allele burdens below 2% (Table 2).

Concerning the mutant allele burden, the JAK2V617F percentage was higher in platelet RNA, than in granulocytes in the two patients with the *JAK2* mutation (Table 2). These results are in agreement with our previous work comparing *JAK2*V617F allele burden in platelets and granulocytes.^6^ Regarding *MPL*W515L, the allele burden was higher in platelet RNA in two of the three mutated patients (Table 2).

Since the detection of a molecular marker is a key element in ET diagnosis and, moreover it may provide additional information regarding the clinical outcome, studies in RNA obtained from platelets and/or granulocytes might be useful in those patients lacking a molecular marker in DNA from granulocytes. To the best of our knowledge, this is the first study evaluating the mutational status of *JAK2*, *MPL* and *CALR* in platelets from TN ET patients. Our results show that 3 out of 35 (8.6%) TN ET patients presented a driver molecular marker when the analysis was performed in platelets.

To gain further insight in the molecular profile of the TN cases we performed targeted NGS mutational analysis in granulocytes from 29 out of the 35 patients included in the study. Overall, we detected 14 additional mutations in 8 (27.6%) patients. Targeted sequencing of *JAK2* and *MPL* showed the presence of mutations in exon 4 of the *MPL* gene in 4/29 (13.8%) patients (Table 2). In three cases the mutations affected the amino acid Ser204: p.S204P (*n*=2) and p.S204F (*n*=1), previously described by other groups.^7, 8^ In the fourth patient a p.P222S was detected which has not been previously reported. We did not find any *JAK2* mutation variant. In addition, mutations in *TET2* (*n*=5), *CBL* (*n*=2), *SF3B1* (*n*=1) and *SH2B3* (*n*=1) were observed (Table 2). These results support the molecular complexity and heterogeneity of TN ET patients.

Interestingly, as shown in Table 2, a somatic mutation in either driver or non-driver genes was detected in all patients who presented monoclonal haematopoiesis assessed by *HUMARA* analysis (6/25, 24%).

In summary, our results reinforce that TN ET patients represent a heterogeneous group of patients in whom the performance of molecular analysis in platelets together with targeted sequencing by NGS techniques provide evidence of clonal hematopoiesis in one-third of patients.

## Figures and Tables

**Table 1:** Clinical and biological characteristics in 35 patients with triple-negative essential thrombocythaemia

Age (years)^a^	58 (9–86)
Gender (M/F)	May-30
Haemoglobin (g/l)^a^	137 (109–166)
Haematocrit (%)^a^	41 (34.2–51.8)
Platelet count (× 10^9^/l)^a^	669 (493–2700)
Leucocyte count (× 10^9^/l)^a^	8.5 (5–22.7)
Clonality by HUMARA^b^, *n* (%)	6 (24)
*Cardiovascular risk factors*	
Smoking, *n* (%)	7 (20)
Hypertension, *n* (%)	14 (40)
Dyslipidaemia, *n* (%)	12 (34.3)
Diabetes mellitus, *n* (%)	6 (17.1)
Thrombosis before diagnosis, *n* (%)	6 (17.1)
Thrombosis during follow-up, *n* (%)	4 (11.4)
Major bleeding before diagnosis, *n* (%)	0 (0)
Major bleeding during follow-up, *n* (%)	4 (11.4)

Haematological and clinical information was collected at diagnosis; information regarding major thrombosis and haemorrhage included events after diagnosis.

a Median (range).

b Informative in 25 female patients.

**Table 2:** Molecular abnormalites detected in 11 triple-negative essential thrombocythaemia patients

*Patient*	*JAK2 p.V617F allele burden (%)*^a^			*MPL p.W515L allele burden (%)*			*Additional mutations by NGS in DNA from GR (allele burden)*	*HUMARA*
	*RNA platelets*	*RNA GR*	*DNA GR*	*RNA platelets*^b^	*RNA GR*^b^	*DNA GR*^c^		
1	20	0	1.85	2.16	0	0	ND	Clonal
2	16	10	1.8	12	33	Positive	ND	NA
3	0			26	15	Positive	ND	NA
4	0			0			*MPL* p.Pro222Ser (42.32%)	Clonal
5	0			0			*MPL* p.Ser204Pro (46.94%)	Clonal
6	0			0			*TET2* p.Gln958Ter (20.17%)	Clonal
							*TET2* p.Leu1880Pro1 (15.2%)	
							*CBL* p.Leu380Pro (2.79%)	
7	0			0			*MPL* p.Ser204Phe (25.69%)	Clonal
8	0			0			*MPL* p.Ser204Pro (37.45%)	Clonal
							*MPL* p.Arg592Gln (30.56%)	
							*SF3B1* p.His662Asp (3.44%)	
9	0			0			*TET2* p.Gln790Ter (20%)	No clonal
10	0			0			*TET2* p.Ser1848Ter (44.74%)	NA
							*SH2B3* p.Trp364Ter (41.47%)	
							*CBL* p.Val363Gly (3.69%)	
11	0			0			*TET2* p.Ala1196Asp (2.41%)	NA

Abbreviations: GR, granulocytes; NA, not available.

a JAK2 V617F was determined by allele-specific real time PCR.

b Determined by NGS.

c Determined by CAST-PCR.
